# Effective delivery of the anti-mycobacterial peptide NZX in mesoporous silica nanoparticles

**DOI:** 10.1371/journal.pone.0212858

**Published:** 2019-02-26

**Authors:** Erik Tenland, Alexander Pochert, Nitya Krishnan, Komal Umashankar Rao, Sadaf Kalsum, Katharina Braun, Izabela Glegola-Madejska, Maria Lerm, Brian D. Robertson, Mika Lindén, Gabriela Godaly

**Affiliations:** 1 Department of Microbiology, Immunology and Glycobiology, Institute of Laboratory Medicine, Lund University, Lund, Sweden; 2 Inorganic Chemistry II, Ulm University, Ulm, Germany; 3 MRC Centre for Molecular Bacteriology and Infection, Department of Medicine, Imperial College London, London, United Kingdom; 4 Department of Clinical and Experimental Medicine, Faculty Medicine and Health Sciences, Linköping, Sweden; University of Padova, Medical School, ITALY

## Abstract

**Background:**

Intracellular delivery of antimicrobial agents by nanoparticles, such as mesoporous silica particles (MSPs), offers an interesting strategy to treat intracellular infections. In tuberculosis (TB), *Mycobacterium tuberculosis* avoids components of the immune system by residing primarily inside alveolar macrophages, which are the desired target for TB therapy.

**Methods and findings:**

We have previously identified a peptide, called NZX, capable of inhibiting both clinical and multi-drug resistant strains of *M*. *tuberculosis* at therapeutic concentrations. In this study we analysed the potential of MSPs containing NZX for the treatment of tuberculosis. The MSPs released functional NZX gradually into simulated lung fluid and the peptide filled MSPs were easily taken up by primary macrophages. In an intracellular infection model, the peptide containing particles showed increased mycobacterial killing compared to free peptide. The therapeutic potential of peptide containing MSPs was investigated in a murine infection model, showing that MSPs preserved the effect to eliminate *M*. *tuberculosis in vivo*.

**Conclusions:**

In this study we found that loading the antimicrobial peptide NZX into MSPs increased the inhibition of intracellular mycobacteria in primary macrophages and preserved the ability to eliminate *M*. *tuberculosis in vivo* in a murine model. Our studies provide evidence for the feasibility of using MSPs for treatment of tuberculosis.

## Introduction

Antimicrobial peptides (AMPs) have gained interest as potential host directed therapeutic strategies against various infections. Defensins comprise one of the largest groups of host defence peptides and are the best studied in infection models [1]. These cysteine-rich, cationic peptides act through disruption of microbial membranes, although they may have additional host-related immune-modulating activities [2, 3]. Peptides are potentially easily degradable and need to be protected in order to safely reach the site of infection where they can exercise their mode of action [4]. To overcome these challenges, peptides can be delivered efficiently by encapsulating them in carrier systems, such as nanoparticles. Nanoparticles can also be designed to allow sustained drug release from the matrix which could have an impact on the treatment time. In this context, mesoporous, amorphous silica nanoparticles (MSPs) represent a highly promising drug delivery platform. The development of MSPs for theranostic applications have been extensively reviewed [5–8], with most research concentrated on the therapeutic effect of small molecular drugs loaded into MSPs; the function of MSPs for intracellular infections is largely unexplored.

Mesoporous silica exhibits variable pore size, making it compatible with the varying sizes of peptide-based drug molecules. Therapeutic molecules inside the porous matrix are efficiently shielded against enzymatic degradation and the particle size can be tuned in the range of 50 nm to 2 μm, allowing the particle size to be adjusted to the requirements of a given route of administration [9, 10]. For maximal bio-distribution of the directed therapy, MSP characteristics can be fine-tuned with regard to size, shape and surface chemistry, to facilitate passage across biological membranes [11, 12]. Recently, MSPs have been used for the delivery of macromolecular hydrophilic drugs, in the form of siRNA, which highlights the potential of MSNs for peptide delivery [13, 14].

Tuberculosis (TB) remains a major health concern with 10 million new cases per year. *Mycobacterium tuberculosis* (*Mtb*) has the capacity to successfully survive within various host environments by changing its metabolic state and avoiding host immune defence mechanisms by intracellular growth. As a result, TB is challenging to treat, requiring lengthy chemotherapy with multi-drug regimens. The duration of treatment is long, varying from six months for drug susceptible infection to more than two years for multi-drug resistant TB [1]. The complexity of infection and the increase in multi-drug resistant TB has stressed the need for the development of new treatment possibilities, and AMPs offer opportunities in this context [2, 3]. We have previously identified a novel candidate, NZX, with anti-mycobacterial activity [15]. NZX is capable of killing clinical and multi-drug resistant strains of *Mtb* at therapeutic concentrations. Furthermore, this peptide substantially reduced bacterial load in the lungs of infected mice after five days of treatment. In this study, we demonstrate that MSPs increase NZX intracellular delivery and preserve its ability to eliminate mycobacteria.

## Methods

### Chemicals

Tetramethylorthosilicate (TMOS, purum, ≥98%, Fluka Analytical/Sigma Aldrich), cetyltrimethylammonium bromide (CTAB, ≥98%, Sigma-Aldrich), (3-aminopropyl) trimethoxysilane (APTMS, 97%, Aldrich Chemistry), methanol (MeOH, technical, VWR), ethanol (EtOH, 99.5% denatured with 1% MEK, VWR, sodium hydroxide (NaOH, ≥98%, Sigma Aldrich). Carboxyethylsilanetriol sodium salt was purchased from GmbH & Co. KG (Karlsruhe, Germany) and Atto488-NH2 from ATTO-TEC GmbH (Siegen, Germany). Phosphate-buffered saline (PBS, Life Technologies), sodium chloride (NaCl, reagent-grade, Merck KGaA), sodium bicarbonate (NaHCO3, reagent-grade, Merck KGaA), potassium chloride (KCl, reagent-grade, Merck KGaA), sodium phosphate monobasic monohydrate (NaH2PO4·H2O, = 99.99%, Merck KGaA), calcium chloride dihydrate (CaCl2·2H2O, purum, Merck KGaA), sodium sulfate (Na2SO4, = 99%, Merck KGaA), tri(hydroxymethyl)aminomethane (TRIS, reagent-grade, Merck KGaA), sodium acetate trihydrate (C2H3NaO2·3H2O, purum, Merck KGaA), sodium citrate dihydrate (C6H5Na3O7·2H2O, reagent-grad)e, Merck KGaA), dipalmitoylphosphatidylcholine (DPPC, Avanti Polar Lipids), 2-[4-(2-hydroxyethyl)-1-piperazinyl]ethanesulfonic acid (HEPES, Merck KgaA) and Milli-Q water. All chemicals were used as received. For the simulated lung fluid (SLF), we used the same composition as previously described [16].

### Electron microscopy

Morphologies and dimensions of the silica nanoparticles were obtained using a Hitachi S-5200 Scanning electron microscope (SEM) (Hitachi, Tokyo, Japan) operated at 20 kV.

Primary macrophages containing MSPs were fixed with 2.5% glutaraldehyde (Fluka, Germany), and postfixed in 2% aqueous osmium tetroxide (Fluka, Germany). The samples were then dehydrated in graded series of 1-propanol and block stained in 1% of uranyl acetate and embedded in Epon (Fluka, Germany). Ultrathin sections (80 nm) were cut on a Leica microtome E, contrasted with 0.3% lead citrate and imaged in a JEOL 1400 TEM (JEOL, Tokyo, Japan) at an accelerating voltage of 120 kV.

### Particle characterization

Nitrogen adsorption and desorption isotherms were measured at -196°C using a Quadrasorb-SI (Quantachrome Instruments, Boynton Beach, USA), and the pore size distributions were calculated using an NLDFT equilibrium kernel developed for silica (Quantachrome). The hydrodynamic size of the particles and the corresponding ζ-potentials in the absence and presence of peptide (in 10^−3^ M KCl) were determined by dynamic light scattering at a scattering angle of 173° using a Zetasizer Nano ZS (Malvern Instruments, Malvern, UK) setup. Measurements were performed in triplicate at 25°C.

### Peptide

The peptide NZX was manufactured by solid phase peptide synthesis, followed by cyclisation of three natural occurring di-sulphide bonds and purification by sequential chromatography steps (PolyPeptide Laboratories AB, Limhamn, Sweden). The purity (>97%) of the peptide was confirmed by high- performance liquid chromatography.

### NZX adsorption and release

For the adsorption isotherms the different silica particles (5 mg/mL) were incubated in PBS buffer solutions (pH 7.4) containing varying peptide concentrations for 1 hour. Afterwards the particles were separated by centrifugation and washed twice with PBS. The peptide concentration in the supernatant was measured using a fluorescamine assay. For the fluorescamine assay 90 μL of supernatant was mixed with 10 μL of fluorescamine solution (5 mg/mL in DMSO) for 5 min. After 5 min the fluorescence was measured at 465 nm using an excitation wavelength of 360 nm. NZX concentration was calculated from a calibration curve obtained using six different calcitonin concentrations.

The peptide release experiments were performed in simulated lung fluid at 0.5 mg/mL particle concentration at 37°C. At given time-points aliquots were taken and the free peptide concentration was measured after centrifugation using a fluorescamine assay.

### Atto488 attachment

To couple the Atto488-NH2 via amide bonds to the particles under mild conditions, the carboxy groups on the particles were first converted into NHS-esters and implemented with Atto488-NH2. Therefore EDC (9.6 μmol/mg) and NHS/HEPES (322.4 mM; 9.9 μmol/mg) were added to a dispersion of carboxy functionalized particles in HEPES buffer solution (pH7.2; 25mM) (14.0 mg/mL) and rotated for 30 min. Afterwards, a mixture of Atto488N amine in DMSO (1.2 mM; 1.2 nmol/mg) was added and stirred for additional 30 min. After 4 h the particles were centrifuged and washed twice with water and twice with ethanol before drying for 24 h in vacuo at room temperature [17].

### Bacteria

*Mycobacterium bovis* (BCG) Montreal strain containing the pSMT1 *luxAB* reporter plasmid was prepared as previously described [18]. Briefly, the mycobacteria were grown in Middlebrook 7H9 culture medium, supplemented with 10% Albumin/Dextrose/Catalase (ADC; Becton Dickinson, UK) and hygromycin (50 mg/mL, Roche, UK), the culture was dispensed into vials, glycerol was added to a final concentration of 50%, and the vials were frozen at -80°C. Prior to each experiment, a vial was defrosted, added to 10 ml of 7H9/ADC/hygromycin medium, and incubated with shaking for 72 h at 37°C. Mycobacteria were centrifuged for 10 min at 3000 g, washed two times and re-suspended in sterile PBS. The bacteria were quantified based on luminescence by adding 0.1% Decanal and measuring relative luminescence units in a luminometer (TrisStar, Berthhold Technologies, Germany).

For murine TB experiments, we used *Mtb* H37Rv with known expression of the surface lipid phthiocerol dimycocerosate (PDIM) (a kind gift from Christophe Guilhot, Institut de Pharmacologie et de Biologie Structurale (IPBS), Toulouse, France). The strain was cultured to mid-log phase in Middlebrook 7H9 culture medium, supplemented with 0.05% Tween 80, 0.2% glycerol and 10% oleic acid-albumin-dextrose-catalase (OADC) enrichment (Becton Dickinson, Oxford, UK).

For the cell count toxicity experiment, *Mtb* H37Rv (ATCC 27294) transformed with a Live-Dead reporter plasmid [19] was used for infecting primary human macrophages. The bacteria were grown to mid-log phase at 37°C in Middlebrook 7H9 medium (BD Biosciences, San Diego, CA, USA) with 0.05% Tween-80, 0.05% glycerol and albumin-dextrose-catalase enrichment (ADC, Becton Dickinson) in the presence of 50μg/ml hygromycin B (Sigma-Aldrich, St Louis, MO) as a selective antibiotic. The bacteria were passaged as 1:9 in the medium and incubated for one more week before use in experiments.

### Cell culture

Human venous blood mononuclear cells were obtained from healthy volunteers using a Lymphoprep density gradient (Axis-Shield, Oslo, Norway) according to the manufacturer’s instructions. To obtain pure monocytes, CD14 micro beads were applied to the cell suspension, washed and passed through a LS-column according to manufacturer’s description (130-050-201, 130-042-401, Miltenyi Biotec, USA). The monocytes were counted (Sysmex), diluted in RPMI 1640 supplemented with 5% FCS, NEAA, 1 mM Sodium Pyruvate, 0.1 mg/ml Gentamicin (11140–035, 111360–039, 15710–49, Gibco, Life Technologies) and 50 ng/ml GM-CSF (215-GM, R&D systems) and seeded in 96-well plates (10^5^/well) for a week to differentiate into macrophages. Infection experiments were performed in RPMI 1640 without Gentamicin.

Human monocytic (THP-1) cells (TIB-202, ATCC, USA) were cultured in RPMI-1640 cell culture medium with 10% fetal bovine serum (FBS) and Antibiotic-Antimycotic antibiotic (Thermo Scientific).

### Minimum inhibitory concentration (MIC)

For MIC comparison between free peptide and peptide loaded in particles, we used resazurin microtiter assay (REMA). Briefly, *Mycobacterium bovis* BCG were seeded in 96-well plates in equal numbers. NZX or NZX-loaded MSP particles were added at final concentrations of 3.2, 6.3 or 12.5 uM NZX (75, 150 or 300 ug/ml particle) and the cultures were incubated at 37°C, 5% CO_2_. Resazurin (1:10 v/v, PrestoBlue Cell viability reagent, Thermo Scientific) was added to untreated control. We used 1:10 and 1:100 diluted control samples as growth controls. The MIC was determined as the peptide concentration where there was no color change to pink while the 1:100 or 1:10 had turned pink, i.e. the lowest concentration that inhibited more than >90% or >99%respectively. The reported MICs are all 99% inhibition.

To measure intracellular MIC, primary human macrophages were prepared as described above. The cells were infected for 24 hours with BCG at a multiplicity of infection (MOI) 10:1 after which the cells were washed three times with PBS. Cells were treated with NZX (3.2–12.5 μM), particles loaded with NZX (MSPs, concentration 75, 150 or 300 μg/ml) or empty particles. The cells were thereafter incubated for one week in 37°C, 5% CO_2_. To determine the level of intracellular bacteria, the cells were washed with PBS, followed by lysis with mammalian-protein extraction reagent (M-PER, Thermo Scientific) for 15 minutes. Decanal was added to a final concentration of 0.1%, and relative luminescence units (RLU) were measured with a TriStar luminometer (TrisStar, Berthhold Technologies, Germany).

### Uptake of particles

Primary macrophages and THP-1 cells were incubated with fluorochrome-conjugated particles (MSP-ATTO488) at concentrations ranging from 25–300 μg/ml. After 0.5-3hours hours of incubation, the cells were detached and the samples analyzed with flow cytometry (Acurin, Becton Dickinson, Oxford, UK). The median fluorescence intensity (MFI) and percentage of cells with a positive fluorescent signal compared to the control (untreated cells) were determined on 5000 gated single cells.

### Time kill assay

MSP released NZX was collected and the concentration was determined using SDS-PAGE. NZX at final concentrations 3.2 uM, 6.3 uM or 12.5 uM was added to BCG in 96 well plates. An additional NZX solution was prepared the same day from powder and added as a control at a final concentration of 3.2 uM. Decanal (0.1% n-decyl aldehyde, Sigma) was added once a day for three days in triplicate wells for each condition. Bioluminescence was measured as relative luminescence units (RLU) with a TriStar luminometer (Berthold Technologies, Germany).

### Confocal microscopy

Primary monocytes were seeded on chamber slides and differentiated to macrophages as described above. MSP-ATTO488 were added at final concentration of 5 or 50 μg/ml and incubated with the cells in 37°C, 5% CO_2_. After 2 hours, live cells were studied in an LSM 510 Meta confocal microscope (Zeiss, Germany).

### Cytotoxicity

Primary human macrophages were prepared as described above and cytotoxicity was measured with ATPlite (Perkin-Elmer) or MTT assay. Empty MSP particles were added at concentrations of 10, 25, 75, 150 or 300 μg/ml and incubated for one week at 37°C, 5% CO_2_. For ATPlite, lysis solution was added followed by 5 min shaking, addition of substrate solution, another 5 min of shaking and incubation for 30 minutes in the dark. Luminescence was measured in a Tecan Infinite F200 reader (Labsystems Multiskan MS, USA). For the MTT assay, 500 μg/ml MTT was added and the plate was incubated for 1 hour at 37°C. Supernatant was removed and the cells were lysed with DMSO. Absorbance at 500 nm was measured with a Tecan Infinite F200 reader.

Infection of primary human macrophages was done with a protocol modified from a previously described method [20, 21]. Briefly, macrophages and *Mtb* H37Rv expressing m-Cherry [19] were mixed in a tube at a MOI of 1 and seeded in 384-well plates. To measure toxicity in infected cells, MSP (37.5, 75, 150 or 300 μg/ml) was added to infected macrophages. After 6 days of incubation, the infected cells were fixed with paraformaldehyde, stained with nuclear stain DAPI and analyzed using ImageXpress (Molecular Devices).

### Murine treatment model

All animal procedures were performed under the license issued by the UK Home Office and in accordance with the Animal Scientific Procedures Act of 1986. Six to eight-week-old female BALB/c mice (Charles River Ltd, UK) were maintained in biosafety containment level 3 (BSL3) facilities at Imperial College London, London, United Kingdom according to institutional protocols [22]. The mice were kept in cages of five animals, with food and water ad libitum. The animals were monitored daily for signs of activity and grooming. Weekly weights with 18% weight loss, or other signs of illness, would trigger a humane endpoint. Veterinary advice was available at all times.

Mice were infected with 1x10^3^ CFU/ml of *M*. *tuberculosis* H37Rv via the intranasal route (control group (n = 15, plus 3 mice to check bacterial numbers implanted in the lungs on day 2), NZX group (n = 5), NZX-MSP (n = 5) and rifampicin group (n = 5). Recognised Schedule 1 methods were used for euthanasia. Two days after infection, three control mice were euthanized to determine the actual dose implanted in the lungs. Five mice from the control group were euthanized prior to the start of treatment in order to determine the bacterial load in the lungs. Following 19 days of infection, the NZX groups were treated for five consecutive days with 0.83 mg NZX (33 mg/kg), NZX-MSP (0.83 mg NZX in 5 mg MSPs) or rifampicin (20 mg/kg), diluted in 50 μl PBS by intra-tracheal administration. The control group received 50 μl PBS by the same route. Following treatment, mice were culled, and the lungs were aseptically removed. The lungs were homogenized in PBS containing 0.05% Tween-80, serially diluted and plated on Middlebrook 7H11 agar plates supplemented with 0.5% glycerol and 10% OADC. The number of CFU from all mice was enumerated 21 days later.

### Statistics

For MIC, intracellular MIC and cytotoxicity, individual experiments were normalized to the average of the negative control. Graphs represent the means of 3 (Cytotoxicity) or 5 (MIC and intracellular MIC) independent experiments. One-way ANOVA followed by multiple comparisons with Dunnets correction was used to calculate the statistical significance in Graph Prism 6 (version 6.0f). Significance was accepted at *p<0.05, **p<0.01 or ***p<0.001.

### Study approval

The animal studies have been approved (PPL 70/7160 and 70/8653) by the Local Animal Welfare and Ethical Review Board (London, UK). The Local Ethical Review Board Dnr 2011/403 and 2014/35 approved the donation of blood from human volunteers for the *in vitro* studies (Lund, Sweden), and the Lund district court approved the control animal studies (Dnr M 7–15). The blood for monocyte isolation for the toxicity analysis was donated by healthy volunteers after receiving information about the study and given their verbal consent (Local Ethical Review Board Dnr 2011/403 and 2014/35). No personal data was collected from the volunteers and the blood was pooled for the isolation of the monocytes. For the intracellular assays, human donor blood was purchased from the blood bank of Linköping University hospitals and blood donors gave written informed consent for research use of the blood.

## Results

### Characterization of MSPs

**Fig 1:**
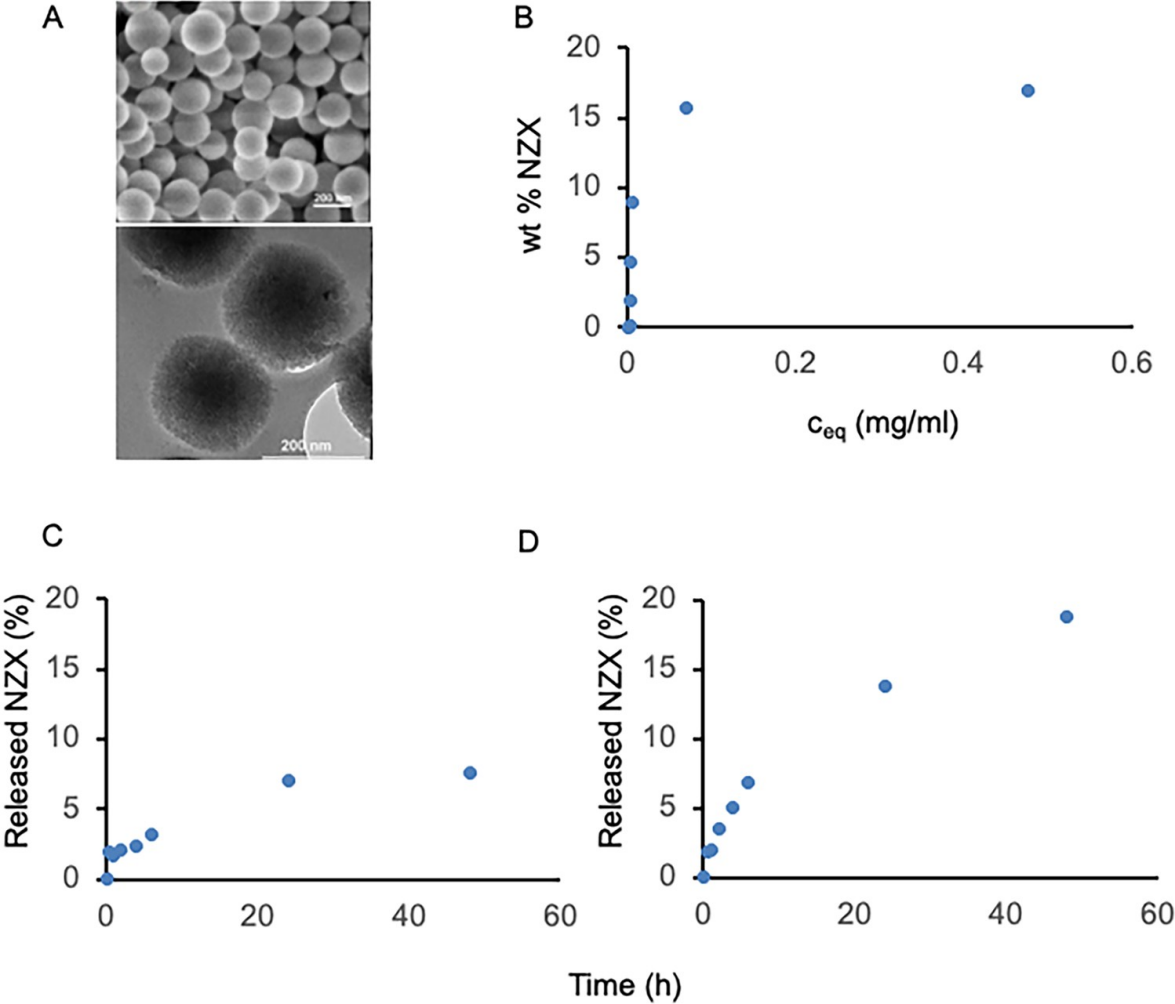
Characterization of MSPs. The synthesis of MSPs and adsorption efficacy of the peptide. (A) SEM image of MSPs with a mean diameter of ~200 nm. (B) NZX adsorbed strongly to the MSPs. (C-D) Release analysis of adsorbed NZX from MSPs as studies in PBS (C) and SLF (D).

MSPs with a mean diameter of about 200 nm were synthesized according to published procedures, as detailed in the methods section. This particle size was chosen in order to enable fast cellular internalization of the particles. Representative electron microscopy images of the particles are shown in Fig 1A.

The mean pore size of the particles was about 3 nm as determined by nitrogen sorption analysis [23]. The BET surface area was 1070 m^2^/g, the pore volume 0.8 mL/g and the zeta potential value measured in water (1 mM KCl, pH 5.5) was -26 mV, in good agreement with previously published values. Thus, the particles exhibit a high porosity, narrow pore size distribution, and a strongly negative surface charge.

### Strong adsorption of NZX to MSPs

The adsorption of NZX onto MSPs was carried out in PBS buffer (pH = 7.2). The adsorption isotherm is shown in Fig 1B. The adsorption isotherm is very steep, indicative of strong attractive interactions between the cationic NZX and the anionic MSPs as expected. A plateau at about 17% wt NZX is seen, which agrees with the loading levels reported for cationic peptides of comparable size [10, 23]. This high value clearly indicates that the majority of the NZX is adsorbed inside the pore system of the MSPs. We note that the strong peptide adsorption allows for an easy formulation of NZX-MSP conjugates, where the conditions can be chosen so that a minimum amount of free peptide is present in solution.

### MSPs releases NZX *in vitro*

The release kinetics of NZX from the NZX-loaded MSPs was studied in either PBS buffer or in simulated lung fluid (SLF, pH = 7.4) at a particle concentration of 5 mg/ml having an NZX-loading of 17% wt. The particle concentration was chosen to be far above the saturation concentration of silica in order to minimize effects related to particle dissolution, and also in order to increase the sensitivity of the peptide quantification in solution. While the PBS buffer solution was lipid-free, the SLF contained 0.02% w/v of the lung relevant lipid dipalmitoylphosphatidylcholine, DPPC. A very slow release of the NZX is observed in PBS, reaching 7.6% of the initially loaded NZX after 48 h, corresponding to an NZX concentration of 14.1 μM (0.062 mg/mL) (Fig 1C). This is still below the value seen in the adsorption isotherm shown in Fig 1B for the same total NZX concentration, C_0_ = 228 μM (1mg/mL), which corresponds to a c_eq_ of 16.2 μM (0.071 mg/mL). Thus, the release in PBS is still below the adsorption equilibrium value and suggests that the adsorption kinetics is much faster than the desorption kinetics in PBS. A clearly faster release with little or no burst release was observed in SLF + 0.02 w/v% DPPC, reaching 18.8% at the same time point (Fig 1D). We ascribe the faster release observed in SLF + 0.02 w/v% DPPC compared to PBS to competition with DPPC for adsorption sites on the silica [24], lowering the attractive NZX-silica interactions, as the pH, ion strength, and the ion composition differences between PBS and SLF are minor.

### MSP released peptide is fully active

**Fig 2:**
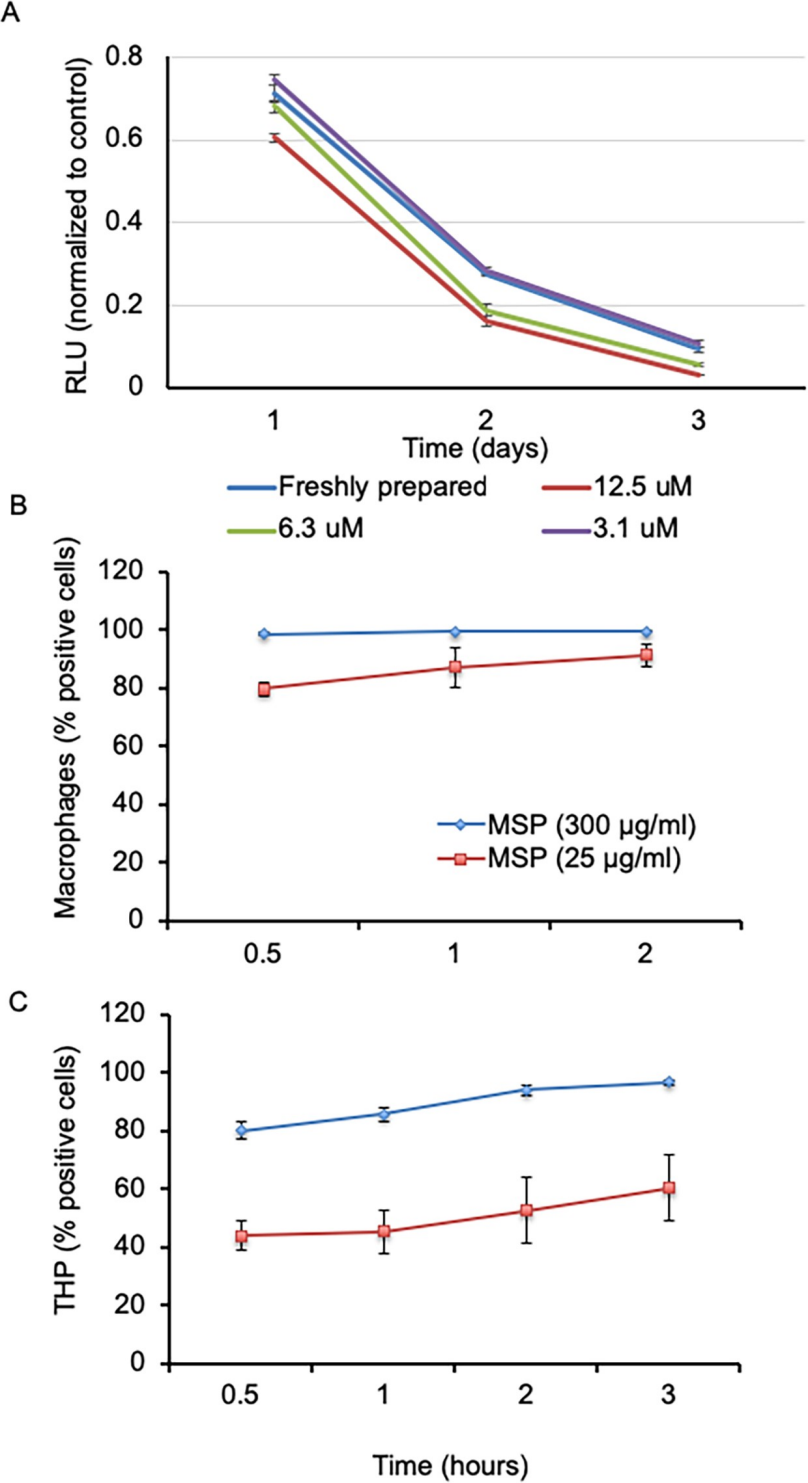
MSPs preserve NZX function and are readily taken up by macrophages. Macrophage uptake of MSPs and functional analysis of MSP-released NZX. (A) Time-kill assay comparing MIC values of freshly prepared NZX and MSP-released NZX showing that the antimycobacterial function was preserved. (B) Kinetic analysis of MSP uptake into primary macrophages and into (C) THP-1 monocytic cells. MSPs were efficiently taken up by both cells, with more rapid uptake by the primary cells.

The loading and release of NZX from MSP particles could potentially alter the antimicrobial function of the peptide. To investigate if this occurs, we performed minimum inhibitory concentration tests on BCG to compare the ability to inhibit growth between free peptide and MSP adsorbed peptide. Resazurin microtiter assays showed identical MIC at 3.2 uM for both formulations (representative image shown in the S1 Fig). In addition, we investigated if the processes of adsorption and release of NZX by the MSPs could affect the antimycobacterial function of the peptide. This was studied by investigating NZX released from MSPs, followed by preparation of solutions with known concentrations. The activity of the released NZX was then compared to that of free NZX in a time kill assay on BCG. The inhibition kinetics of BCG was the same for both 3.2 μM solutions (Fig 2A), indicating no altering in the antimycobacterial inhibition by adsorption and release from MSPs.

### Macrophages efficiently take up NZX loaded MSPs

In order to evaluate the cellular internalization kinetics of the MSP, fluorescent (Atto488-conjugated) particles were synthesized using carboxylic acid-functionalized MSP-COOH of the same size and charge as the all-silica MSPs discussed above. Primary macrophages and THP-1 cells, a monocyte cell line, were used to investigate the uptake of MSPs into cells. Fluorochrome-conjugated MSP (25 and 300 μg/ml) was added to the cells and the fraction of particle-positive cells determined by flow cytometry. The particles were taken up by a larger percentage of cells at both concentrations (Fig 2B and 2C). The uptake was more efficient in primary macrophages, with most of the cells displaying particle uptake after half an hour at the high 300 ug/ml MSP concentration (Fig 2B), compared to 80% uptake for THP-1 monocytes (Fig 2C). For both macrophages and THP-1 cells, the percentages were lower, 80% and 40% respectively at the lower 25 ug/ml concentration. In all cases, the percentage of positive cells increased with time.

### Macrophage stores MSPs in vesicles

**Fig 3:**
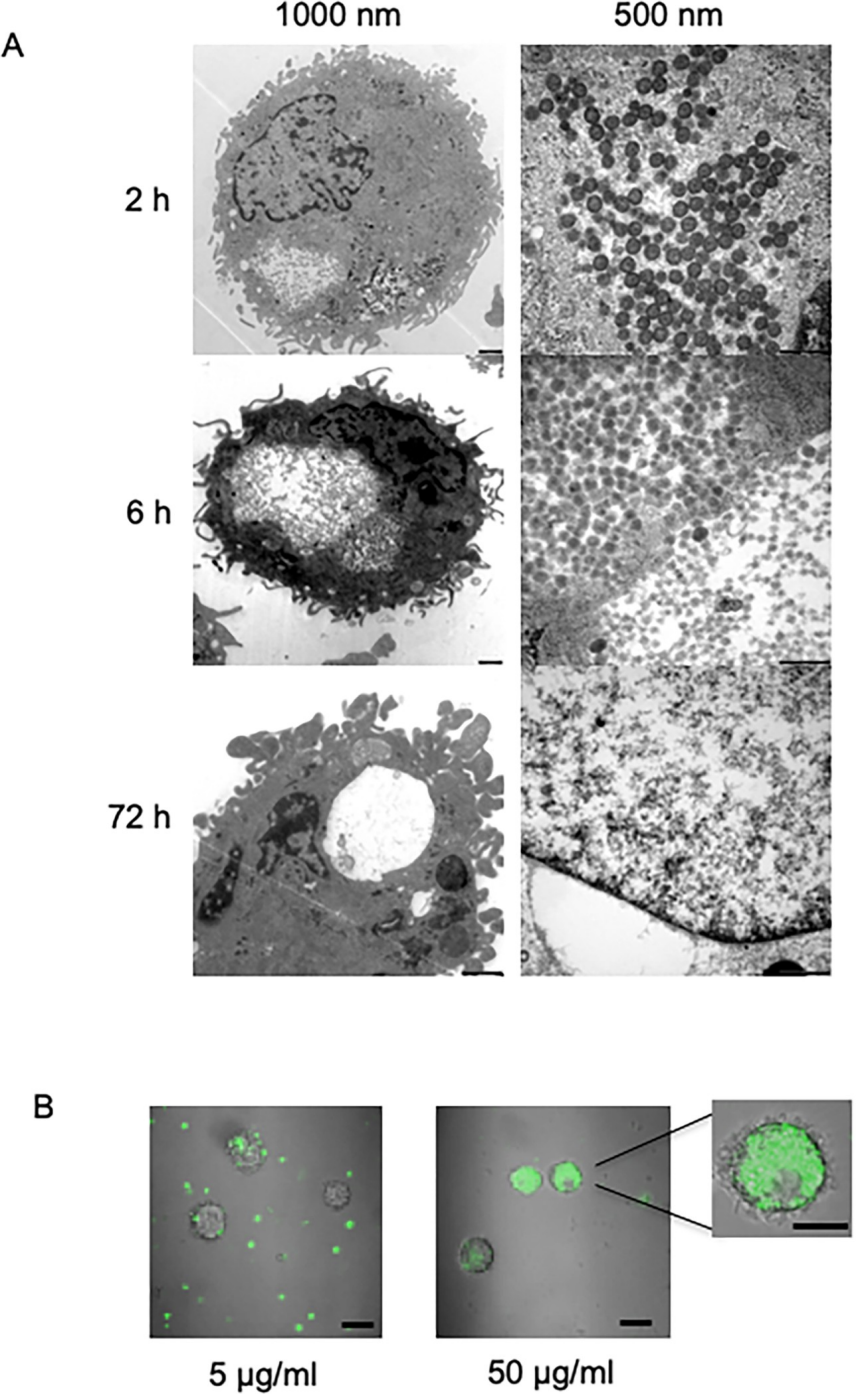
Macrophages store MSPs in vesicles. (A) TEM visualization of MSPs inside primary macrophages. Macrophages were observed to concentrate the internalised MSPs in vesicle-like structures. Over time, degradation of the internalized particles was observed. (B) Confocal microscopy of Atto-488 conjugated MSPs in primary macrophages showing concentration-dependent uptake to the cytosol after 2 hours.

To study the uptake and intracellular breakdown of particles, primary macrophages were incubated with 300 μg/ml MSPs, collected after 2–72 hours and studied with TEM (Fig 3A). Large collections of intact particles could be found in vacuoles at early time points. Intracellular breakdown of particles became apparent after 72 hours.

To further investigate and visualize the uptake of MSP in primary macrophages we used confocal microscopy. Primary macrophages were incubated for 2 hours with either 5 or 50 μg/ml of fluorescent particles. At both concentrations, macrophages engulfed the particles (Fig 3B), however this was more pronounced at 50 μg/ml.

### MSPs induced dose dependent toxicity

**Fig 4:**
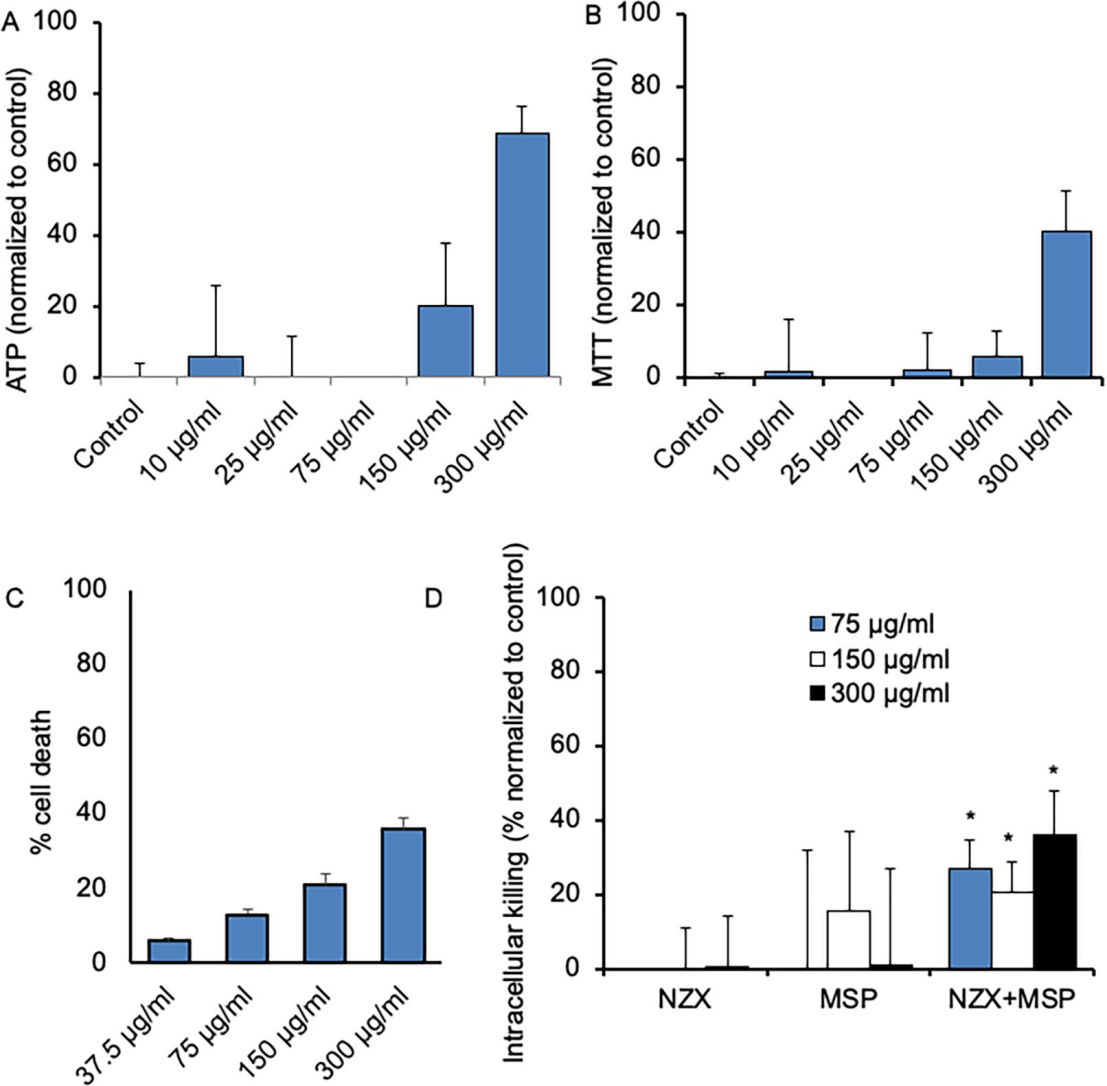
MSPs increased intracellular killing of mycobacteria. Toxicity was studied using different assays. (A) ATPlite and (B) MTT assays showed dose dependent toxicity MSP. (C) Cell count of *M*. *tuberculosis* H37Rv infected primary macrophages treated with MSPs also revealed dose dependent toxicity. (D) Time-kill assay of intracellular MIC showing that MSPs containing NZX showed a significant increase in mycobacterial inhibition compared to free peptide (mean of five separate experiments, *p<0.05).

We used different methods to study toxicity of the MSPs. MTT and ATPlite assays showed a variable, but mostly dose dependent toxicity (Fig 4A and 4B). In addition, by measuring cell count in *Mtb* H37Rv infected primary macrophages we observed that MSPs induced a dose-related toxicity (Fig 4C).

### Peptide containing MSPs increased intracellular bacterial killing

The intracellular bacterial killing efficiency at therapeutically relevant peptide concentrations was studied with free NZX peptide and the MSP loaded NZX in an MIC assay. Analysis of infected primary macrophages revealed that cells treated with NZX loaded MSPs showed significantly more inhibition compared to free peptide (Fig 4D).

### MSPs containing peptide are effective *in vivo*

**Fig 5:**
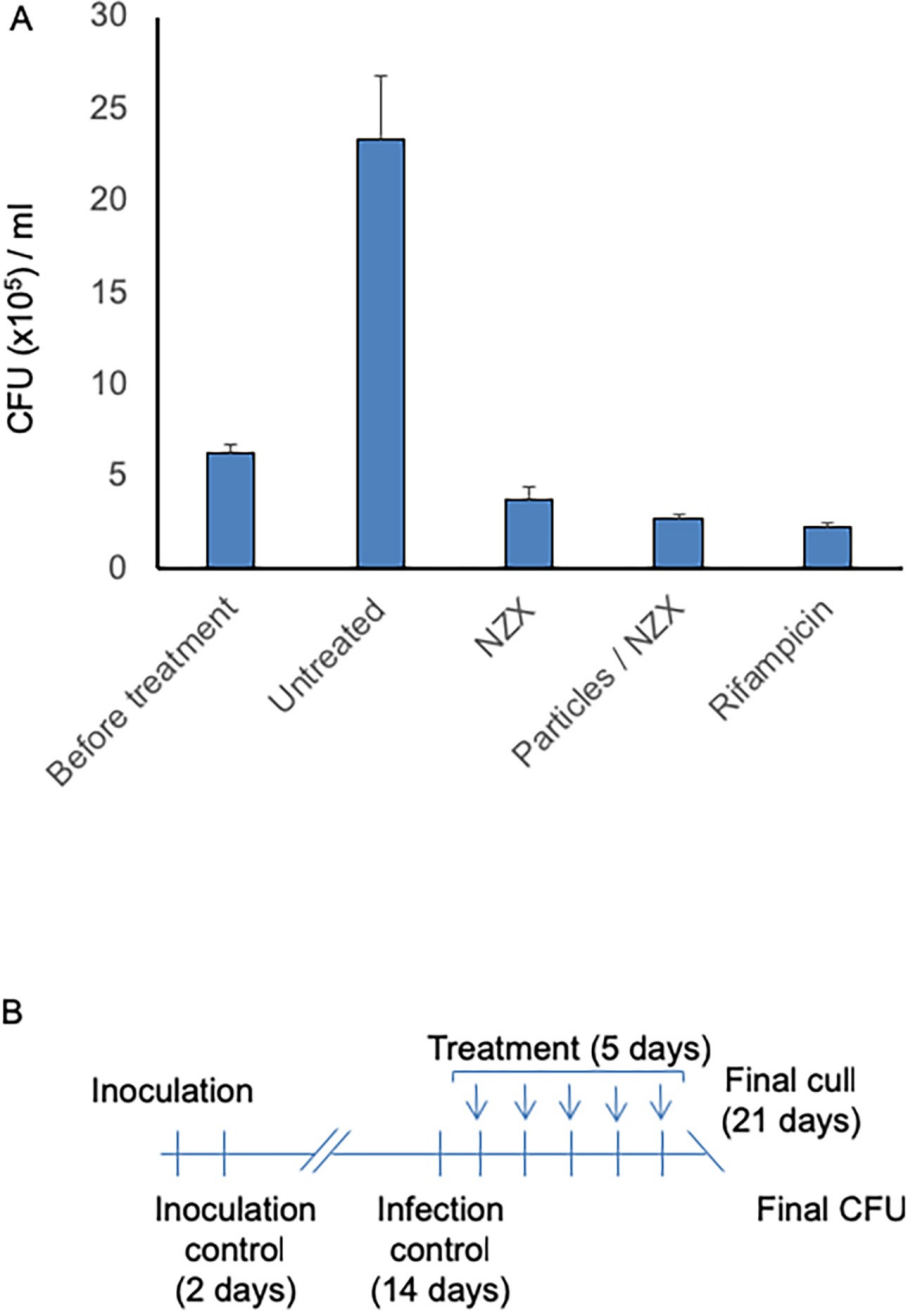
MZX-MSPs function in an *in vivo* model of TB. The ability of NZX MSPs to kill *Mtb* H37Rv in a murine model was analysed. (A) Daily endotracheal administration of free NZX, MSPs containing NZX, or rifampicin for five days reduced lung CFU. Data are presented as mean ± sd. P-values were calculated by ANOVA with Tukey’s correction and results compared to infection control are shown (**p<0.01, ***p<0.001). (B) Schematic representation of experimental setup for murine pulmonary TB with *Mtb* H37Rv.

Bactericidal activity experiments were performed in a murine TB model with *M*. *tuberculosis* H37Rv [22] (Fig 5). The mean bacterial dose implanted in the lungs, measured two days after infection, was 520 ± 32 CFU/ml. After 14 days of infection, there was an increase in lung CFU to 6.3x10^5^ CFU/ml. The animals received five doses of NZX, MSP-NZX or rifampicin by intra-tracheal administration, while one control group was left untreated. Compared to the untreated control, we found a reduction in CFU by 84% (Fig 5, p = 0.0079) in the lungs of mice treated with NZX, an 88% decrease in CFU (p = 0.0159) in the lungs of mice treated with MSP-NZX, and a reduction by 90% (p = 0.0079) in the rifampicin treated group. Comparing all three treatment groups with the untreated group, we found significant differences (p = 0.0004). We found no significant difference between NZX, MSP-NZX and rifampicin treated groups.

## Discussion

We have designed mesoporous nanoparticles containing the antimicrobial peptide NZX at therapeutic concentrations. These MSPs were non-toxic to mammalian cells at therapeutic concentrations but showed increased intracellular anti-mycobacterial activity. In a murine model of TB, we showed that NZX containing MSPs significantly decreased the bacterial load, at levels comparable to rifampicin treatment. The MSPs were synthesized with a mean diameter of ~200 nm in order to enable fast cellular internalization of the particles by alveolar macrophages [25]. For MSPs, a recent study showed that HeLa cells favoured a particle size of 50–120 nm [26]. In our study, the particles were taken up efficiently by the majority of the cells, with the most efficient engulfment by primary macrophages. This observation agrees with previous studies reporting that primary cells have increased uptake efficiency compared to cell-line [26, 27].

The majority of the NZX is adsorbed inside the pore system of the MSPs. Similar adsorption isotherms have been observed for the cationic peptides LL-37 and calcitonin [23], which indicates that also in the case of NZX multidirectional NZX-silica interactions are responsible for the strong peptide adsorption to the MSPs. The strong peptide adsorption allows for an easy formulation of NZX-MSP conjugates, in which conditions can be chosen to minimise the amount of free peptide present in the solution. SLF is based on the composition of human respiratory tract lining fluid and suitable for studying the biopharmaceutical properties of inhaled medicines [28]. The observed gradual release of NZX in SLF suggests that prolonged release could also occur in the lung. Importantly, MSP containment of the peptide did not alter its function as the released peptide had preserved antimycobacterial capacity.

Macrophage uptake of nano-sized particles proceeds mainly via pinocytosis, specifically clathrin-mediated endocytosis, and is responsible for internalization of particles ranging in size from 100–350 nm. After internalization the material is stored in vesicles that are programmed to fuse with the lysosomal compartments for degradation of the contents [29]. We observed a large collection of intact particles in vacuoles shortly after uptake that slowly broke down over three days, possibly as a function of infused free radicals to the lysosome. This is important as the degradation and clearance of particles is important to avoid their accumulation in the body. In our previous study we observed that the peptide is bactericidal as a single dose of 1.6 or 3.2 μM could kill mycobacteria [15]. Here we could see that the degradation time frame did not affect peptide function, as we observed improved intracellular killing with peptide loaded MSPs compared to free peptide. Furthermore, in the animal model we observed a tendency that MSPs containing NZX were more effective than free peptide in controlling the infection, although there was no statistical difference between the groups. A chronic model of infection could probably clarify these results.

Intracellular bacteria shelter themselves from many antibiotics by residing in human cells; in tuberculosis, treatment targeting intracellular bacteria key. NZX is an antimicrobial peptide with proven activity against *M*. *tuberculosis in vitro* and *in vivo* [15]. In this study, loading NZX into MSPs increased the inhibition of intracellular mycobacteria in primary macrophages and preserved the ability to eliminate *M*. *tuberculosis in vivo* in a murine model, supporting their future therapeutic application.

## Supporting information

S1 Fig Resazurin microtiter assay was used for MIC comparison between free peptide and peptide loaded in particles. Representative image identical MIC at 3.2 uM for both formulations.
(TIF)
