# Supplementary figures and images for: Effective delivery of the anti-mycobacterial peptide NZX in mesoporous silica nanoparticles
Source: PLoS One. 2019 Feb 26;14(2):e0212858. doi: 10.1371/journal.pone.0212858 (PMC6391042; doi:10.1371/journal.pone.0212858)

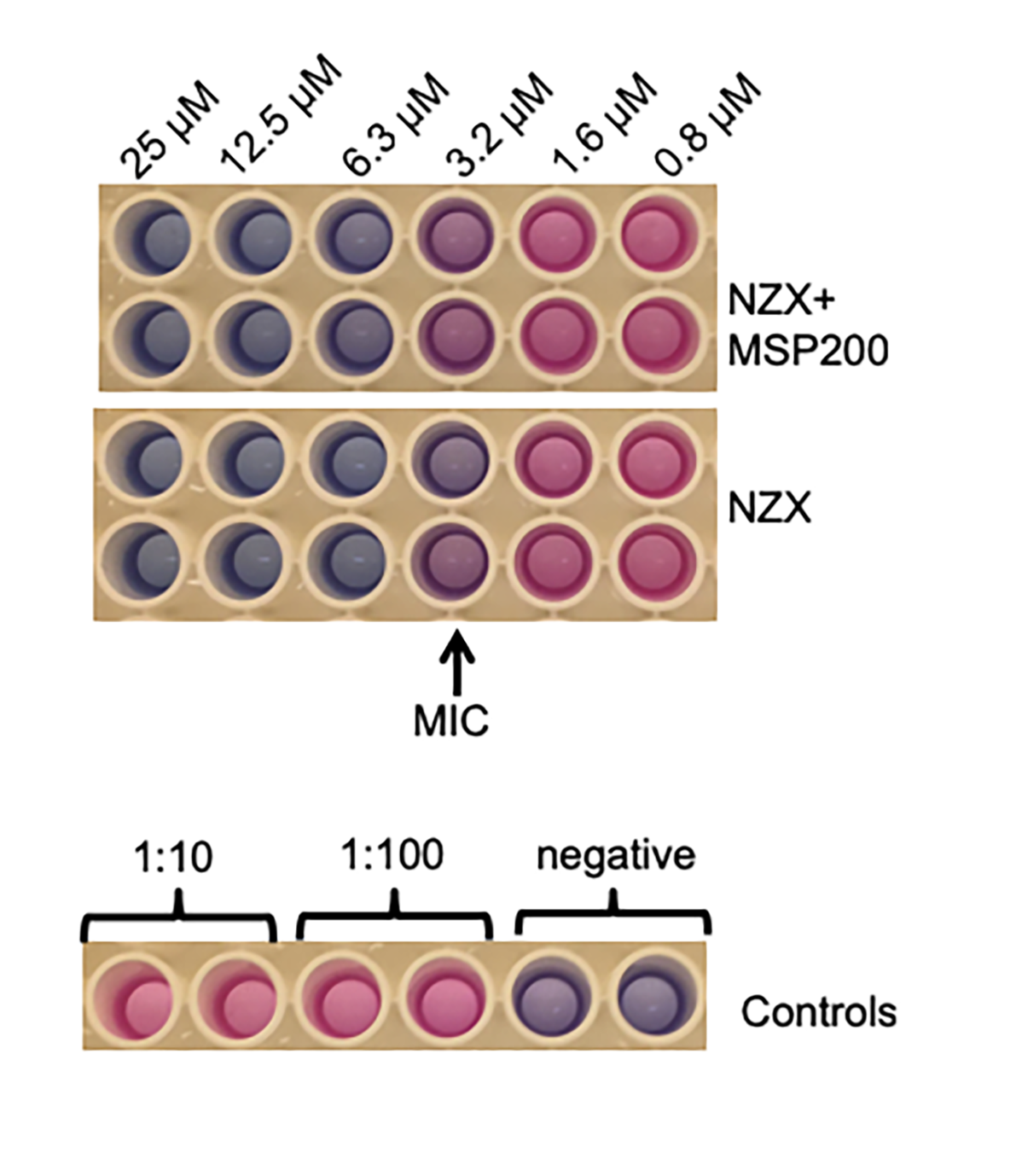

Supplement: S1 Fig — Representative image identical MIC at 3.2 uM for both formulations. (TIF) [file pone.0212858.s001.tif]
